# Treatment Outcomes of Diabetic Patients With Erectile Dysfunction Prescribed High-Dose Tadalafil

**DOI:** 10.7759/cureus.34812

**Published:** 2023-02-09

**Authors:** Antony Condina, Tatiana Lykina

**Affiliations:** 1 Pharmacy, Oceania University of Medicine, Samoa, AUS; 2 Allergy and Immunology, Oceania University of Medicine, Samoa, AUS

**Keywords:** treatment outcomes, high dose tadalafil, erectile dysfunction, diabetes, alternate day dosing

## Abstract

*Objective*: To assess the treatment outcome of diabetic patients with erectile dysfunction who are prescribed an alternate daily high dose of tadalafil over a 120-day treatment period.

*Methods*: This was a single-site, retrospective, observational study of 63 diabetic men with erectile dysfunction prescribed an alternate daily dose of 30mg of tadalafil between January 1, 2021, and December 31, 2021. Treatment outcomes accessed medication compliance, adverse drug reactions, and patient treatment satisfaction at 60- and 120-days treatment.

*Results*: Mean age of patients was 58.3 years and included patients who suffered from comorbidities ranging from hypertension (54.0%), dyslipidemia (52.3%), and depression (9.5%). At 60 days in the study, 69.8% were satisfied and continued the treatment. However, at the end of the 120-treatment period, a low number of men (17.5%) were satisfied with the treatment and therefore did not remain on the treatment protocol. These patients reported a lack of medication dose efficacy (86.5%), non-compliance with treatment as prescribed (65.4%), and adverse drug reactions (30.8%) as reasons for discontinuing treatment. None of the identified patient demographics were significantly associated with 120-day continuous treatment. Similarly, the odds ratio derived from the logistic regression did not demonstrate an association between the selected variables and the outcome of 120-day continuous treatment retention.

*Conclusion*: This retrospective case series study found that 82.5% of diabetic patients were not satisfied with treatment with alternate dosing of 30mg tadalafil to treat their ED at the end of the 120-day treatment period suggesting an alternative treatment plan.

## Introduction

Erectile dysfunction (ED) is defined as a consistent alteration in the quality of erections that adversely affected the patient’s satisfaction with sexual intercourse [1]. ED is associated with medical conditions such as diabetes and hypertension, lifestyle factors, and medical treatments (beta blockers, thiazides, antidepressants, and prostate cancer surgery) and has been recognized as an early indicator of cardiovascular disease [2]. In Australia, ED is experienced by about one in five men over the age of 40 years [3]. The prevalence of ED with diabetes is much higher at 35%-75% [4-10]. In diabetes, hyperglycemia is considered the main factor for macro and microvascular complications, increasing sexual dysfunction through oxidative stress and impairing erectile and endothelial function. Autonomic neuropathy may also play a role in the pathogenesis, as do associated arteriosclerosis, hypertension, and hyperlipidemia [11].

Current first-line treatments include the phosphodiesterase 5 inhibitors (PDE5Is) sildenafil, vardenafil, avanafil, and tadalafil [1]. These drugs increase the intracellular cyclic guanosine monophosphate concentrations in cavernous tissue leading to the relaxation of arterial and trabecular smooth muscle and increasing arterial inflow and the rigidity of penile erection [12]. The efficacy of PDE5Is in non-diabetic men is about 60%-70% [1]. However, efficacy is lower in diabetic men due to impaired endothelium-derived factors in penile arteries and underlying endothelial dysfunction [13-16]. This may explain why ED is more refractory to treatment in diabetic men.

Previous studies have demonstrated men with diabetes; and ED can benefit from daily low doses of tadalafil (2.5 and 5mg) as an alternative to on-demand treatment of 5 to 20mg [12, 16]. A steady state of tadalafil is reached after five days of daily administration, with a plasma concentration that is roughly 1.6 times higher than that of a single dose [17]. Higher doses may be required in men who require greater serum concentrations for therapeutic effect by higher peak levels. However, higher doses can also be associated with adverse reactions such as headache, flushing, dyspepsia, nasal congestion, and dizziness [18-19].

Presented is a retrospective, observational case series of Australian diabetic patients with ED presenting to a single men’s health clinic in Australia and subsequently prescribed an alternate daily dose of 30mg tadalafil. The treatment outcome of these patients was assessed over a 120-day treatment period.

## Materials and methods

**Figure 1:**
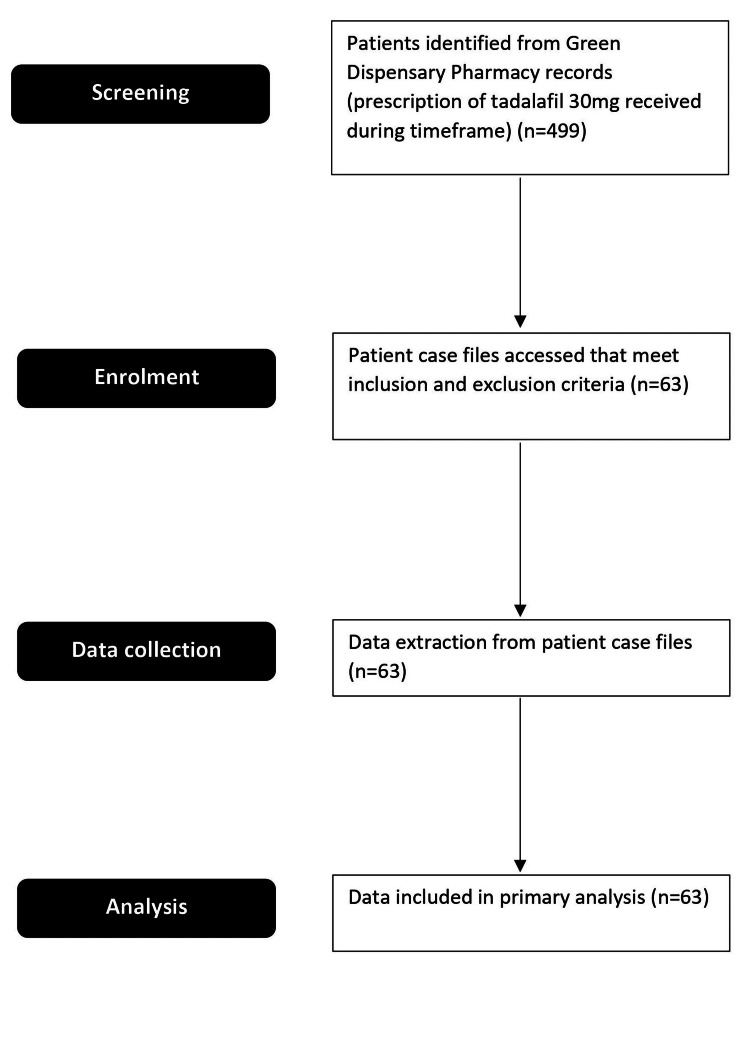
Process for identifying eligible patients

The process for identifying patients in the study is summarized in Figure 1. Green Dispensary Pharmacy South Australia’s electronic dispensing database was accessed to identify patients for whom a prescription for 30mg tadalafil capsule was prescribed during the period between January 1, 2021, and December 31, 2021. Of these patients, the patient case notes from Men’s Health Clinic Australia were accessed that met the inclusion and exclusion criteria. Each man was dispensed 30 capsules of extemporaneously compounded tadalafil 30mg capsules (60 days supply) at each visit. Patients were instructed to take one capsule alternate day continuously. Patients were interviewed by a practice nurse at each visit. Treatment outcome accessed medication compliance, adverse drug reactions, and patient treatment satisfaction. Treatment compliance was defined as taking at least 21 capsules over each visit (70% of required doses for a total of 42 capsules out of the 60 capsules dispensed), which was confirmed with engagement with the clinic and from the pharmacy dispensing history [16]. Measures of treatment satisfaction in this study were based on the two Global Assessment Questions (GAQ): ‘Has the treatment you have been taking improved your erectile function?’ (GAG1) and if ‘yes,’ then ‘has the treatment improved your ability to engage in sexual activity?’ (GAG2). These questions were presented to patients during their visits. Adverse drug reactions included common adverse reactions such as headache, flushing, dyspepsia, nasal congestion, and dizziness.

Inclusion criteria: Eligible men were ≥ 18 years old with a history of diabetes, having insulin treatment or receiving an oral glucose-lowering agent, diagnosis of ED based on the Sexual Health Inventory for Men (SHIM) questionnaire, and a dispensed prescription for 30mg tadalafil.

Exclusion criteria: Men were excluded from the study if they had uncontrolled hypertension, unstable coronary disease, renal and hepatic insufficiency, were newly diagnosed with diabetes during the study period or had radical pelvic surgery, penile prosthesis surgery, used penile devices, hormonal therapy, or concurrent use of other PDE5Is or other ED medications, and nitrate therapy.

All data extraction was performed by the primary researcher (AC). Ethics approval for this study was obtained from the relevant Human Research Ethics Committee (OUM 22-0419AC).

Data analysis

Standard descriptive statistics were used to present the demographic and other characteristics of patients. Univariate assessment of categorical and continuous variables was performed initially, using Fischer’s exact test. Logistic regression was subsequently performed on selected variables. An alpha level of 0.05 was selected for statistical significance.

All statistical analyses were performed using Minitab® statistical software (Penn State University).

## Results

Patients

Participant demographic and clinical characteristics are presented in Table 1. The sample (n=63) had a mean patient age of 58.3 years with a mean body mass index of 30.6 kg/m². Eight (12.7%) were current smokers, and 11 (17.4%) were current consumers of alcohol. Most patients had comorbidities ranging from hypertension (54.0%), dyslipidemia (52.3%), and depression (9.5%). These patients were prescribed an oral anti-diabetic drug 58 (92.1%), insulin 16 (25.4%), antihypertensive drug 27 (42.9%), or antihyperlipidemic drug 31 (49.2%). All patients suffered from ED for more than 12 months. Out of the 63 patients, 24 (38.1%), 17 (27.0%), and 22 (34.9%) reported having mild, moderate, and severe ED, respectively.

**Table 1:** Participant demographic and clinical characteristics

Number of Patients, (%)	63 (100%)
Age (years) (mean ± SD)	58.3 ± 10.9
Body mass index (kg/m^2^) (mean ± SD)	30.6 ± 5.0
Current smokers (%)	8 (12.7%)
Current consumers of alcohol (%)	11 (17.4%)
Comorbidities (excluding diabetes) (%)	
Hypertension	34 (54.0%)
Depression	6 (9.5%)
Dyslipidemia	33 (52.3%)
Medication (%)	
Oral anti-diabetic drug	58 (92.1%)
Insulin	16 (25.4%)
Antihypertensive drug	27 (42.9%)
Antihyperlipidemic drug	31 (49.2%)
Erectile dysfunction duration ≥ 12 months (%)	63 (100%)
Erectile dysfunction severity at baseline (SHIM) score (%)	
Mild	24 (38.1%)
Moderate	17 (27.0%)
Severe	22 (34.9%)

Treatment outcomes

**Figure 2:**
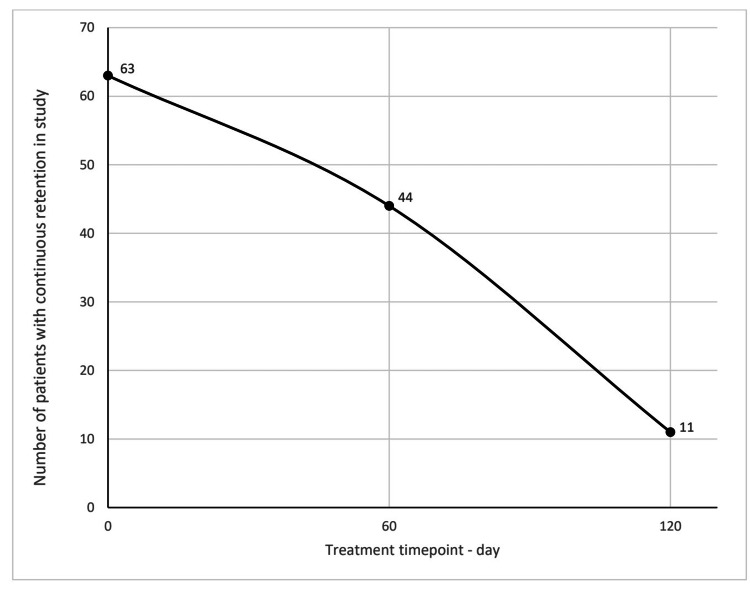
Continuous retention of patients in study at 60 and 120 days

**Figure 3:**
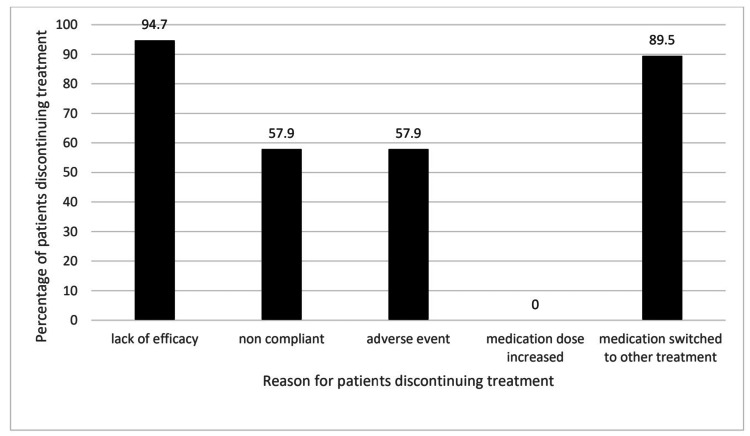
Reasons for patient non-retention at 60 days

Patients were assessed at 60 and 120 days, respectively, investigating treatment outcomes. Of the 63 patients in the study, 44 (69.8%) and 11 (17.5%) were satisfied and remained in the treatment at 60 and 120 days, respectively (Table 2 and Figure 2). At 60 days, of the 19 patients that did not continue the treatment as prescribed, 18 (94.7%) stated that a lack of efficacy was a reason not to continue, 11 (57.9%) patients did not continue due to non-compliance with the medication as prescribed, 11 (57.9%) patients experienced adverse drug reactions and had to stop treatment. These patients experienced an overlap of reasons for discontinuing treatment. Medication dosage was not increased in these 19 patients, and 17 (89.5%) patients were switched to other treatments (Figure 3).

**Table 2:** Patient treatment satisfaction

	60 days	120 days
Treatment satisfaction based on GAQ1 yes responses	44 (69.8%)	11 (17.5%)
Treatment satisfaction based on GAQ2 yes responses	44 (69.8%)	11 (17.5%)

**Figure 4:**
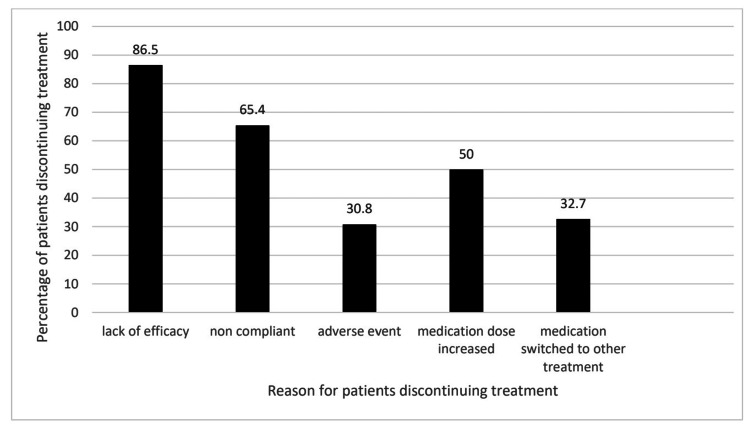
Reasons for patient non-retention at 120 days

At 120 days, of the 52 patients that did not continue the treatment as prescribed, 45 (86.5%) stated that a lack of efficacy was a reason not to continue, 34 (65.4%) patients did not continue due to non-compliance with the medication as prescribed, 16 (30.8%) patients experience adverse drug reactions and had to stop treatment. Medication dosage was increased in 26 (50.0%) patients, and 17 (32.7%) patients were switched to other treatments (Figure 4). These 52 patients that were accessed at 120 days included the 19 patients accessed at 60 days. Many of the reasons for discontinuing treatment overlapped.

Data showed that based on univariate analysis (Fischer’s exact test), none of the identified patient demographics were significantly associated with 120-day continuous treatment. The severity of ED (mild 63.64%, moderate 18.2%, severe 18.2%) was also not significant (P = 0.174). Similarly, the odds ratio derived from the logistic regression did not demonstrate an association between the selected variables and the outcome of 120-day continuous treatment retention (Table 3).

**Table 3:** Fischer’s exact test for the association of patient characteristics and 120-day continuous treatment retention

Variable	Continuous retention of patients in study at 120 days (n = 11)	P-value	Odds Ratio (95% CI)
Hypertension	8 (72.7%)	0.32	1.80 (0.43, 7.61)
Depression	1 (9.1%)	0.72	0.94 (0.01, 8.94)
Dyslipidemia	8 (72.7%)	0.12	2.88 (0.69, 12.08)
Current smokers	1 (9.1%)	0.57	0.64 (0.07, 5.83)
Current consumers of alcohol	3 (27.3%)	0.29	2.06 (0.45, 9.49)

## Discussion

This study is the first to describe the treatment outcomes of diabetic patients with ED over a 120-day treatment period using alternate day dosing of 30mg tadalafil (high dose). Current prescribing guidelines for ED recommend tadalafil 10-20mg orally at a time before sexual activity or 2.5 to 5mg once daily [20]. However, there is no guidance on dosing based on ED severity or other comorbidities. Doses of up to 40mg daily have been used in patients with pulmonary arterial hypertension and are well tolerated [21]. Even though 30mg of tadalafil is not routinely used in the clinic continuously for ED, our study shows that higher doses or other treatments may be required in diabetic patients with refractory ED or patients unsatisfied with sexual performance.

There were only 11 patients out of the 63 samples (17.5%) that continued the treatment for 120 days. There are several reasons that these diabetic patients did not obtain a favorable treatment outcome at 120 days. Sexual stimulation is required for nitric oxide to be released from nerves and endothelial cells directly into the penis to elicit an erection. Since erectile/endothelial damage and autonomic neuropathy may impair this pathway, likely, diabetic patients with comorbidities may not sustain the level of cGMP required to elicit smooth muscle relaxation and, therefore, an erection [1,14].

All the diabetic patients suffered from ED for more than 12 months before they presented to the clinic. A 30mg dose of tadalafil was chosen as these men did not obtain favorable sexual results with the lower approved doses. With the continuous 30mg alternated day dosing, a steady-state concentration of tadalafil would equate to approximately 1.6 times higher than that of a single dose. The relatively high serum concentration from this dosing regimen may have contributed to some of the reasons for the non-retention of treatment at 120 days. Interestingly, the higher serum concentration of tadalafil did not contribute to positive efficacy. This is in contrast to previous work that showed efficacy at 5mg of tadalafil daily [16]. The difference is attributable to the small sample size of our study.

Univariate analysis of the data presented did not demonstrate a statistically significant association between individual participant demographics and the outcome with 120-day continuous treatment. Similarly, the odds ratio derived from the logistic regression did not demonstrate an association between the selected variables and the outcome of 120-day continuous treatment retention. The large association confidence intervals are likely to reflect the effect of small sample sizes.

While the presented case series demonstrated unfavorable outcomes for diabetic patients using alternate day 30mg tadalafil, several limitations frame the presented observations. Patients' self-reported sexual satisfaction at 60 days and 120 days will affect recall bias. The obtained results from this single-site specialized medical clinic may not be generalizable to other general clinics. The severity of the men’s diabetes and duration was not assessed in this study as all men that presented to the single clinic had been previously diagnosed and treated for diabetes by other healthcare professionals not involved in the study. Medication compliance was defined as taking at least 70% of the required prescribed doses between visits. This was based on previous work by Hartzichristou D et al. [16]. 65.4% of patients were not compliant with the prescribed dosing leading to unfavorable outcomes at 120 days.

As an observational case series, this study did not intend to analyze the effectiveness of 30mg tadalafil alternate daily dosing in diabetic patients with ED. Case series are descriptive and do not set out to test hypotheses related to efficacy. Large numbers and effect sizes are needed to make positive observations regarding outcomes in case series. However, case series reports have been recognized as a utility in improving case definition, providing clues, generating hypotheses, and informing follow-up studies [22].

## Conclusions

As diabetes continues to grow more prevalent in the western world, ED in diabetic patients is coincidingly a growing concern. PDEIs have lower efficacy in diabetic men due to impaired endothelium-derived factors in penile arteries and underlying endothelial dysfunction. Therefore, to address the sexual well-being of diabetic patients with ED, the optimal treatment for ED needs to be investigated.

In summary, our retrospective case series study found that 82.5% of diabetic patients were not satisfied with treatment with alternate dosing of 30mg tadalafil to treat their ED at the end of the 120-day treatment period suggesting an alternative treatment plan.
